# Heterochannels Kv(1.1-1.2)_2_ and Their Interactions with Pore Blockers

**DOI:** 10.3390/cells14171364

**Published:** 2025-09-02

**Authors:** Anastasija V. Efremenko, Elena V. Kryukova, Oleg V. Kazakov, Anastasia A. Ignatova, Ivan I. Shmatin, Varvara N. Korabeynikova, Victoria A. Toporova, Sergey A. Yakimov, Mikhail P. Kirpichnikov, Oksana V. Nekrasova, Alexey V. Feofanov

**Affiliations:** 1 Shemyakin-Ovchinnikov Institute of Bioorganic Chemistry, Russian Academy of Sciences, 117997 Moscow, Russia; aefr@mail.ru (A.V.E.); evkr@mail.ru (E.V.K.); kazakov.oleg.v@yandex.ru (O.V.K.); aignatova_83@mail.ru (A.A.I.); shmatinva@gmail.com (I.I.S.); var.kora.3@gmail.com (V.N.K.); toporova@ibch.ru (V.A.T.); sa-yakimov@yandex.ru (S.A.Y.); kirpichnikov@inbox.ru (M.P.K.); onekrasova@ibch.ru (O.V.N.); 2 Faculty of Biology, Lomonosov Moscow State University, 119234 Moscow, Russia

**Keywords:** Kv1.2, Kv1.1, voltage-gated, heterochannel, peptide blocker, fluorescent microscopy, patch clamp, dissociation constant, competitive binding

## Abstract

Heterotetramerization of Kv1.1 and Kv1.2 α-subunits expands the functional diversity of voltage-gated potassium Kv1 channels in the central nervous system (CNS), thus necessitating the study of the properties of these heterochannels, including their interactions with ligands. We report on the expression, electrophysiological, and ligand-binding properties of human heterochannels Kv(1.1-1.2)_2_ formed by dimeric concatemers Kv1.1-Kv1.2 fused with fluorescent protein mKate2 in Neuro-2a cells. Kv(1.1-1.2)_2_ is a low-voltage-activated, highly active, non-inactivating channel with a fast activation rate. Its activation rate and half-maximum activation voltage are similar to that of the Kv1.1 channel, but differ from that of Kv1.2. This suggests that the membrane expression of Kv(1.1-1.2)_2_ may functionally compensate for the absence of membrane presentation of homotetrameric Kv1.1 channels in CNS. Hongotoxin 1 fused with fluorescent protein GFP (HgTx-G) is shown to be a pore-blocking ligand of Kv(1.1-1.2)_2_ with a dissociation constant of 100 pM. Using confocal microscopy and competitive binding assay, HgTx-G and cells expressing Kv(1.1-1.2)_2_, the apparent dissociation constants of the complexes between Kv(1.1-1.2)_2_ and peptides Ce1, Ce4, hongotoxin 1, MeKTx11-1, agitoxin 2, charybdotoxin, and scyllatoxin were evaluated to be 14, 33, 40, 250, 800, and >>3300 pM, respectively. Heterotetramerization of α-subunits has a different effect on the affinity of ligands compared to those for Kv1.1 and Kv1.2 channels.

## 1. Introduction

Voltage-gated potassium channels of the Shaker family (Kv1) play a regulatory role in neuronal excitability by influencing the conduction of action potential and neurotransmitter release [1,2]. Kv1 channels are integral membrane proteins composed of four α-subunits that form a potassium-selective pore. Four of the six transmembrane helices from each α-subunit participate in formation of the voltage-sensing domain, while the other two helices are an integral part of the channel pore. The voltage-sensing domain regulates potassium ion transport through the pore in response to changes in the transmembrane potential using a channel gating mechanism. The essential structural element of the pore is a P-loop that connects transmembrane helices related to the pore domain, maintains the structural integrity of the pore and forms the external binding site for pore blockers. The ligands that block Kv1 channels from the outside are mostly peptide toxins from animal venoms, which are distinguished for their high affinity and selectivity for the target Kv1 channels [3]. Some of these peptides are widely used to study Kv1 channel structure and functions. The N- and C-terminal domains of α-subunits, which are localized in cytoplasm, play structural and regulatory roles. The N-terminal domain governs the assembly of the channel and maintains its stability [4]. It is involved in the N-type inactivation of the channel by blocking the open channel pore from the cytoplasmic side [5]. The C-terminal domain is known to contain structural motifs that govern differential intracellular trafficking and expression of Kv1 channels in the plasma membrane [6]. Eight isoforms of α-subunits of Kv1 channels, Kv1.1-Kv1.8, are known.

The Kv1 channels are abundant in the central nervous system (CNS), where they are present in the form of homo- or hetero-tetramers composed of identical or different isoforms of α-subunits, respectively. Kv1 homo- and hetero-tetramers are characterized by a diverse distribution pattern in the CNS and are clustered in distinct neuronal compartments [2,7], where they form complexes with a number of membrane-associated proteins, such as synaptic channel-clustering proteins SAP97 and PSD-95 [8], contactin-associated protein 2 in the juxtaparanodal region of axon [9], or secreted leucine-rich glioma-inactivated protein 1 in the transsynaptic protein complex [10]. Hetero- versus homo-tetramerization of the Kv1 α-subunits, as well as their interactions with specialized proteins, provides functional diversity of Kv1 channels and localization-dependent tuning of their activity in neurons.

The ability of Kv1 α-subunits to form heterotetramers is determined by the homology of their T1 domains located in the N-terminal region [4]. Although various Kv1 α-subunits readily co-assemble in heterologous expression systems [11,12], only a limited subset of Kv1 heterochannels have been found in the CNS [13,14]. The Kv1.2 α-subunit is present in all Kv1 heterochannels found in the CNS, and its partners are Kv1.1, Kv1.3, Kv1.4, and Kv1.6 α-subunits. The most common types of heterochannels are Kv1.1/Kv1.2 and, to a lesser extent, Kv1.2/Kv1.4. Notably, Kv1.2 and Kv1.4 α-subunits also formed homotetrameric channels. In contrast, in the CNS, Kv1.1, Kv1.3, and Kv1.6 α-subunits have only been detected in the form of heterotetramers. However, in a mouse model of multiple sclerosis, an increased expression of Kv1.1 homotetrameric channels was revealed in demyelinated axons [15].

The composition of heterochannels in vivo was determined by using a sequential precipitation/Western blotting approach with antibodies that are specific for distinct Kv1 α-subunits [13,16,17]. In addition to immunochemical methods, peptide blockers with increased affinity for specific homotetrameric Kv1 channels have been widely used to identify isoforms of Kv1 channels and reveal their localization in various cells and specific subcellular compartments [18,19,20]. Among them are dendrotoxins (DTXs) from snake venom and scorpion toxins, such as TsTx-kα, hongotoxin 1 (HgTx1) and margatoxin, as well as their radio- or fluorescently labeled derivatives [14,19,21,22,23]. However, in these studies, the stoichiometry of α-subunits in natural Kv1 heterochannels could not be determined. Moreover, to explore the properties of heterochannels with different subunit ratios, specific approaches are required.

To study the properties of Kv1 heterochannels with a predefined stoichiometry and position of different α-subunits, it was proposed to sequentially link the genes encoding the required Kv1 α-subunits and express the obtained concatemeric constructs in oocytes or mammalian cells [24,25,26,27,28,29]. The concatemers of Kv1 α-subunits were shown to embed themselves in the plasma membrane and form functional channels. The correct functioning of the Kv1 channels formed by the concatemers has been demonstrated for concatemers Kv1.1-Kv1.1, Kv1.2-Kv1.2, and Kv1.1-Kv1.1-Kv1.1-Kv1.1 [24,27,28,29]. The approach based on the expression of concatenated α-subunits provided data on the electrophysiological properties of heterochannels with different compositions and stoichiometries [15,24,25,26,27,30]. Using concatemers, the study of pharmacological properties of heterochannels was initiated, and affinities of some peptide blockers for these channels were characterized [15,24,25,26,31,32]. Although the number of peptides studied is still very limited, a combination of α-DTX and DTX-k dendrotoxins was identified that helped to differentiate between isoforms of channels composed of Kv1.1 and Kv1.2 α-subunits in neurons. These studies demonstrated that peptide blockers are a promising alternative to Kv1 channel-specific antibodies in studies of the composition, localization, and functional role of various heterochannels.

Kv1 channels serve as targets for various peptide toxins found in the venom of animals such as scorpions, snakes, and sea anemones. To date, most studies have focused on these peptides as ligands for the homotetrameric Kv1 channels. It is time to characterize these peptides with respect to heterochannels with different subunit compositions. Screening of known peptide blockers may potentially reveal selective high-affinity ligands for heterochannels, and/or may help to understand structure–activity relationships in order to design them.

Recently, we have developed a general approach to the engineering of cellular systems for the study of peptide blockers of Kv1 channels [33]. It is based on the fluorescent Kv1 channel expressed in mammalian cells and the fluorescent high-affinity ligand, whose interactions are measured with the laser scanning confocal microscopy and analyzed using an optimized algorithm of the image treatment. Any non-fluorescent peptide can be characterized in this system as a potential ligand of the expressed channels using the competitive binding protocol, and its affinity to the channels can be estimated. This approach was realized for homotetrameric Kv1.1, Kv1.2, and Kv1.3 channels [33,34,35]. In the present study, we report on the extension of this approach to heterochannels Kv(1.1-1.2)_2_ of the 2:2 stoichiometry and their peptide blockers. The expression of dimeric concatemers Kv1.1-Kv1.2 fused with fluorescent protein mKate2, which has a different order of α-subunits, was characterized in Neuro-2a cells, and the electrophysiological properties of the corresponding channels were measured. HgTx1 fused with fluorescent protein GFP (HgTx-G) was shown to be a high-affinity ligand of the Kv(1.1-1.2)_2_ channels. Interactions of the Kv(1.1-1.2)_2_ channels with agitoxin 2 (AgTx2), HgTx1, charybdotoxin (ChTx), scyllatoxin (ScyTx), MeKTx11-1, Ce1, and Ce4 toxins were studied, and the dissociation constants of complexes were evaluated.

## 2. Materials and Methods

### 2.1. Reagents, Recombinant Peptides, and Proteins

GenJector-U transfection reagent was from Molecta (Moscow, Russia). Lyso Tracker Green, NBD-labeled C6-ceramide, ER Tracker Green, and transferrin labeled with Alexa Fluor 488 were from ThermoFisher Scientific (Waltham, MA, USA). Bovine serum albumin and rhodamine 123 were from Merck (Darmstadt, Germany). Oligonucleotide primers (see Section 2.2) were synthesized by Evrogen (Moscow, Russia).

ChTx, AgTx2, HgTx1, ScyTx, MeKTx11-1, Ce1, and Ce4 peptides were obtained according to protocols published previously [36,37,38]. HgTx-G was expressed and purified as described earlier [35].

### 2.2. Expression Plasmids

To construct the gene encoding a fluorescent concatenated dimer mKate2-Kv1.1-Kv1.2 (K-Kv1.1-1.2), a cloning strategy was used, in which the C-terminus of the human Kv1.1 α-subunit was fused end-to-end to the N-terminus of the human Kv1.2 α-subunit via a short KL linker that corresponds to the HindIII restriction site. Here, we followed the design of the α-subunit concatemers proposed earlier [39]. The previously obtained plasmids pmKate2-KCNA1 and pmKate2-KCNA2, which coded for mKate2-Kv1.1 (K-Kv1.1) and mKate2-Kv1.2 (K-Kv1.2) fusion proteins, were used for cloning [34,35]. In these proteins, both human α-subunits Kv1.1 and Kv1.2 are N-terminally tagged with the red fluorescent protein mKate2 and harbor single mutations (S369T in Kv1.1, and S371T in Kv1.2) that improve the surface presentation of the homotetrameric channels [40]. At first, pmKate2-KCNA1stopless was generated by amplification of the *KCNA1* gene lacking TAA stop codon in a polymerase chain reaction (PCR) using a pair of oligonucleotide primers Kcna1-f1 (5′-TTCTCAGATCTATGACGGTGATGTCTGGGGAGAACGT-3′) and Kcna1-Stop-r1 (5′-TCTTCAAGCTTAACATCGGTCAGTAGCTTGC-3′; BglII and HindIII sites are underlined). The obtained DNA fragment was hydrolyzed by BglII/HindIII restriction endonucleases and cloned into pmKate2-KCNA1 plasmid to substitute KCNA1 for KCNA1stopless. The obtained plasmid pmKate2-KCNA1stopless was further used as a vector to produce the KCNA1-KCNA2 dimer. For this, the *KCNA2* gene was amplified in PCR using primers Kcna2-Hind-f1 (5′- CTTCAAGCTTACAGTGGCCACCGGAGACCCAGCAGA-3′) and Kcna2-Sal-r1 (TTCGTCGACTCTCCTGCAG**TTA**GACATCAGTTAACATTTTGGTAATATTC-3′; HindIII, SalGI, and PstI sites are underlined; TAA stop codon is marked in bold) and cloned into HindIII/SalI sites of pmKate2-KCNA1stopless. The resulting pmKate2-KCNA1-KCNA2 plasmid encoded K-Kv1.1-1.2.

To produce a fluorescent concatenated dimer mKate2-Kv1.2-Kv1.1 (K-Kv1.2-1.1), pmKate2-KCNA2stopless was first generated by cloning into BglII/HindIII sites of pmKate2-KCNA2 plasmid a *KCNA2stopless* gene, which was amplified in PCR with primers Kcna2-f1 (5′-TTCTCAGATCTATGACAGTGGCCACCGGAGACCCA-3′) and Kcna2-Stop-r1 (5′-CTTCAAGCTTGACATCAGTTAACATTTTGGTA). Then, the KCNA1 gene was amplified with primers Kcna1-Hind-f1 (5′-CCTCAAGCTTACGGTGATGTCTGGGGAGAACGTGGA-3′) and Kcna1-Sal-r1 (5′-TTCGTCGACTCTCCTGCAG**TTA**AACATCGGTCAGTAGCTTGCTCT-3′) and cloned into HindIII/SalI sites of pmKate2-KCNA2stopless to get pmKate2-KCNA2-KCNA1 plasmid, which was coded for K-Kv1.2-1.1.

The sequences of dimeric *KCNA1-KCNA2* and *KCNA2-KCNA1* genes were confirmed in the created plasmids pmKate2-KCNA1-KCNA2 and pmKate2-KCNA2-KCNA1, respectively, by Sanger sequencing of both strands (Evrogen, Moscow, Russia).

### 2.3. Cells and Experiments with Cells

Mouse neuroblastoma Neuro-2a cells (the Russian collection of cell cultures, the Institute of Cytology RAS, Saint Petersburg, Russia), were grown in Dulbecco’s modified Eagle’s medium DMEM/F12 supplemented with 2 mM L-glutamine (Paneco, Russia) and 10% fetal bovine serum (HyClone, Logan, UT, USA; i.e., a complete medium). Cells were subcultured two times per week.

Cells were seeded (3 × 10^4^ cells per ml, per well) on poly-L-lysine-coated cover glasses in 24-well plates, grown for 24 h, and subjected to transfection with plasmids using GenJector-U reagent. Experiments were conducted 24 h after transfection. The efficiency of transfection was 40–50%.

Protocols for staining lysosomes, mitochondria, endosomes, Golgi apparatus, and endoplasmic reticulum were described previously [33,34].

To analyze the binding of HgTx-G to heterochannels, HgTx-G (0.032–2 nM) was added to cells in the complete medium for 1 h. In the experiments with competitive binding, HgTx-G (0.5 nM) was added to cells, together with the studied peptide (0.05–2 nM HgTx1; 1.25–100 nM ChTx; 0.5–20 nM AgTx2, 2–10 nM MeKTx11-1, 0.063–1 nM Ce1, 0.063–1 nM Ce4, or 1 µM ScyTx), and incubated in the complete medium for 1 h.

Comparison of the ligand binding to the closed and open channels was performed as described previously [34]. Briefly, cells were incubated with different concentrations of HgTx-G (0.03–2 nM) for 1 h in a buffer (20 mM HEPES pH 7.4, 0.9 mM CaCl_2_, 0.5 mM MgCl_2_, and 0.1% bovine serum albumin) supplemented either with 4 mM KCl and 150 mM NaCl (Na^+^ buffer) or with 150 mM KCl (K^+^ buffer).

### 2.4. Confocal Microscopy

Confocal microscopy measurements were carried out with the SP2 laser scanning confocal microscope (Leica Microsystems GmbH, Wetzlar, Germany), using the 63× water-immersion objective (HCX PL APO, NA = 1.2). Axial and lateral resolutions were 0.6 and 0.2 µm.

Fluorescence of proteins labeled with mKate2 was excited at 561 nm and detected in the 680–800 nm region. Fluorescence of HgTx-G was excited at 488 nm and recorded in the 495–540 nm region. The fluorescent probes of various cellular organelles were excited at 488 nm and detected in the 495–540 nm region.

### 2.5. Quantitative Analysis of Interactions Between Heterochannels and Ligands

Quantitative analysis of HgTx-G interaction with heterochannels was performed following the procedure developed previously [33]. Briefly, confocal images of cells were used to calculate the ratio of the fluorescence intensity of HgTx-G bound to heterochannels to the fluorescence intensity of membrane-embedded heterochannels for each measured cell. This ratio was averaged over a sample of measured cells (20–30 cells) to obtain the *R_av_* value and its standard deviation.

Measuring the dependence of *R_av_* on the concentration, *L*, of HgTx-G added to cells and fitting this dependence with equation

*R_av_*(*L*)/*R_m_* = *L*/(*K_d_* + *L*) (1)

the dissociation constant (*K_d_*) of the complexes was estimated. In Equation (1), *R_m_* is the maximal *R_av_* value at the saturation of HgTx-G binding. *K_d_* was estimated in independent measurements, averaged, and presented as mean ± SEM (*n* = 3).

To characterize the affinity of unlabeled peptide for heterochannels, the competitive binding of this peptide and HgTx-G to heterochannels was measured at the constant concentration of HgTx-G (*L*) and increasing concentration (*C*) of the peptide. *R_av_* data were calculated, plotted as a function of *C,* and fitted with the following equation:

*R_av_*(*C*) = *R_av0_*/(1 + *C*/*DC_50_*) (2)

where *DC_50_* is the peptide concentration displacing 50% of HgTx-G from the complex with heterochannels, and *R_av0_* is *R_av_* at *C* = 0.

Further, the Cheng–Prusoff equation was used to calculate the apparent dissociation constant (*K_ap_*) of the complex between the studied peptide and heterochannels:

*K_ap_* = *DC_50_*/(1 + *L*/*K_d_*) (3)

Three independent experiments were performed, and data on *K_ap_* were averaged and presented as mean ± SEM.

### 2.6. Electrophysiology Measurements

Membrane Kv1-related ion currents were recorded at room temperature by the patch-clamp technique, using the standard whole-cell configuration as described previously [34,35].

Briefly, 10 mm diameter coverslips pre-coated with poly-L-lysine with attached Neuro-2a cells were placed 24–48 h after transfection into a chamber containing an extracellular solution (mM)—140 NaCl, 5 KCl, 2 MgCl_2_, 2 CaCl_2_, 10 HEPES, and 10 glucose (pH 7.4)—and perfused continuously. Micropipettes with tip resistance of 4–6 MΩ were filled with the pipette solution containing (mM) 140 KCl, 6 CaCl_2_, 2 MgCl_2_, 2 MgATP, 0.4 NaGTP, 10 HEPES, and 20 BAPTA/KOH (pH 7.3). Patches were routinely held at a holding potential (HP) of −70 mV. Test 200 ms pulses were applied stepwise (20 mV step) with an interval of 5 s, from −70 to +70 mV, returning after each step to HP. Currents were recorded using the EPC-10 amplifier (HEKA Elektronik, Reutlingen, Germany). For experiments, cells, which possess bright red fluorescence and maximal outward currents greater than 2 nA were selected.

The time constant of channel activation (*τ_a_*) was determined by approximating the section of the time dependence of the K+ current, starting from the moment of voltage change until the achievement of maximum current (*I_m_*), with a mono-exponential function:

*I*(*t*) = *I_m_*(1 − exp(−*t*/*τ_a_*)) (4)

Values of *τ_a_* determined for several cells were averaged and presented as mean ± SEM.

To calculate the half-maximum activation voltage (*V_1/2_*), the conductivity (*G*) of the cell was determined using the following formula:

*G* = *I*/(*V − E_K_*) (5)

where *I* is the value of K^+^ current 200 ms after the channel activation, *V* is the activating potential, and *E_K_* is the reversal potential (−80 mV).

The dependence of the normalized conductivity, *G*/*G_m_*, on the applied voltage was approximated using the Boltzmann equation:

*G*/*G_m_* = 1/(1 + exp((*V_1/2_ − V*)/*k*)) (6)

where *G_m_* is the maximum conductivity, and *k* is the slope coefficient.

Values of *V_1/2_* determined for several cells were averaged and presented as mean ± SEM.

## 3. Results

### 3.1. Design of Concatemers K-Kv1.1-1.2 and K-Kv1.2-1.1

Earlier, we constructed tandem dimers K-Kv1.1-1.1 and K-Kv1.2-1.2 and demonstrated that the electrophysiological properties of the channel formed by each of these concatemers were exactly the same as those of the channel formed by corresponding monomeric α-subunits Kv1.1 and Kv1.2 fused with mKate2 [28,29]. These properties were also similar to those reported by other investigators for the corresponding homotetrameric channels, Kv1.1 and Kv1.2. It was also shown that the *K_d_* of the complexes between HgTx-G, a high-affinity fluorescent ligand for the Kv1.1 and Kv1.2 channels [34,35], and the channel formed by each of the concatemers, K-Kv1.1-1.1 and K-Kv1.2-1.2, were equal to the *K_d_* of the complexes between HgTx-G and the corresponding channels formed by monomeric α-subunits Kv1.1 and Kv1.2 fused with mKate2 [28,29]. In all cases, HgTx-G was displaced from the complexes by peptide blocker HgTx1.

These results suggest that the linking of Kv1.1 or Kv1.2 subunits into concatemers using a short dipeptide and fusing them with mKate2 do not alter the properties of the channels formed by these concatemers. Accordingly, in the present work, we decided to use the same approach to combine subunits Kv1.1 and Kv1.2 into the concatenated proteins, K-Kv1.1-1.2 and K-Kv1.2-1.1, assuming that it would not affect the native properties of the Kv(1.1-1.2)_2_ heterochannels. Two concatemers, K-Kv1.1-1.2 and K-Kv1.2-1.1, were constructed to test the dependence of the properties of the Kv(1.1-1.2)_2_ heterochannels on the order of α-subunits in the concatemer.

### 3.2. Expression and Distribution of Concatemers K-Kv1.1-1.2 and K-Kv1.2-1.1 in Neuro-2a Cells

**Figure 1:**
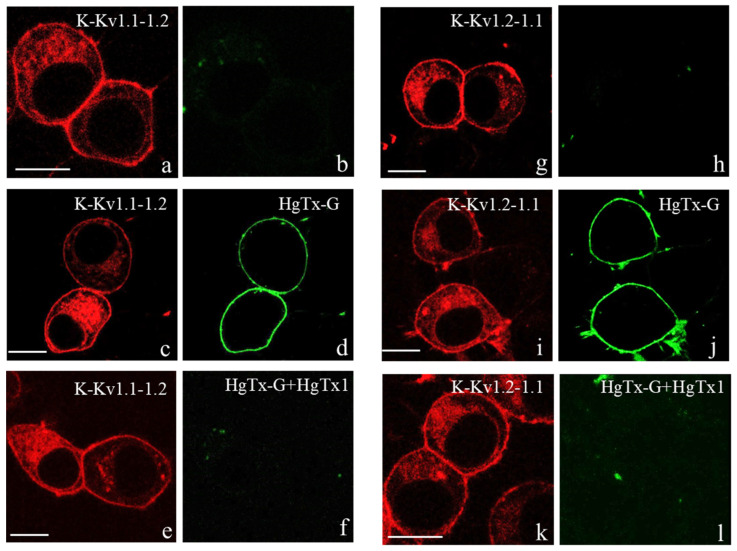
Expression of K-Kv1.1-1.2 and K-Kv1.2-1.1 in Neuro-2a cells and interactions of heterochannels with HgTx-G and HgTx1. (**a**–**l**) Red color shows typical distribution of K-Kv1.1-1.2 (**a**,**c**,**e**) and Kv1.2-Kv1.1 (**g**,**i**,**k**) in cells. Green color shows distribution of fluorescence in the 495–540 nm range in the absence of HgTx-G (**b**,**h**), in the presence of 0.5 nM HgTx-G (**d**,**j**), and in the presence of 0.5 nM HgTx-G and 10 nM HgTx1 (**f**,**l**). Bar is 10 µm.

Heterological expression of K-Kv1.1-1.2 or K-Kv1.2-1.1 in Neuro-2a cells occurs in 40-50% of cells subjected to transfection with the plasmids pmKate2-KCNA1-KCNA2 or pmKate2-KCNA2-KCNA1 and is characterized by similar patterns of intracellular distribution of both proteins (Figure 1). The concatenated constructions form a network-like distribution in the cytoplasm and brightly stain plasma membrane. 

**Figure 2:**
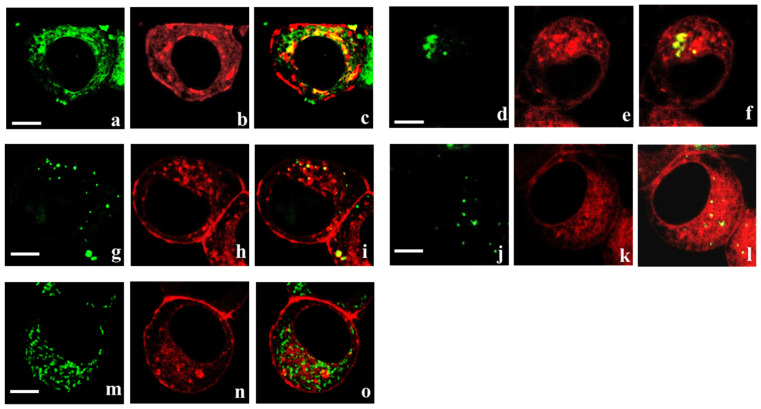
Confocal fluorescence analysis of localization of K-Kv1.1-1.2 in ER (**a**–**c**), Golgi apparatus (**d**–**f**), endosomes (**g**–**i**), lysosomes (**j**–**l**), and mitochondria (**m**–**o**) of Neuro-2a cells. Red color shows distribution of K-Kv1.1-1.2 in cells. Green color shows distribution of endoplasmic reticulum marker ER Tracker Green (**a**,**c**), Golgi apparatus marker NBD-labeled C6-ceramide (**d**,**f**), endosome marker transferrin labeled with Alexa Fluor 488 (**g**,**i**), lysosome marker Lyso Tracker Green (**j**,**l**), or mitochondrion marker rhodamine 123 (**m**,**o**) in cells. Yellow color in the merged images (**c**,**f**,**i**,**l**) indicates the localization of K-Kv1.1-1.2 in endoplasmic reticulum (**c**), Golgi apparatus (**f**), endosomes (**i**), and lysosomes (**l**). No localization of K-Kv1.1-1.2 in mitochondria is observed (**o**). Scale bar is 5 µm.

Analysis of the intracellular distribution of K-Kv1.1-1.2 has revealed its localization in the endoplasmic reticulum and Golgi apparatus (Figure 2a–f), the organelles directly involved in the assembly of ion channels and their transport to plasma membrane. Localization of K-Kv1.1-1.2 in some lysosomes and endosomes was also observed (Figure 2g–l). This suggests a limited participation of membrane-bound K-Kv1.1-1.2 in endocytosis, followed by its sequestration in lysosomes for proteolytic degradation. The presence of K-Kv1.1-1.2 in mitochondria was not observed (Figure 2m–o).

Membrane-embedded K-Kv1.1-1.2 and K-Kv1.2-1.1 are able to bind HgTx-G (Figure 1c,d,i,g). No binding of HgTx-G was observed for untransfected Neuro-2a cells [34,35], as well as for cells transfected with plasmids but not expressing K-Kv1.1-1.2 or K-Kv1.2-1.1 on the plasma membrane (Figure 1c,d,i,g). HgTx1 displaced HgTx-G from the complexes with K-Kv1.1-1.2 and K-Kv1.2-1.1 (Figure 1e,f,k,l). This means the formation of the binding site for peptide blockers by K-Kv1.1-1.2 and K-Kv1.2-1.1 that usually requires the assembly of four α-subunits into the channel. Competition between HgTx-G and HgTx1 indicates that their binding site is reasonably the same.

### 3.3. Properties of Heterochannels Formed by Concatemers K-Kv1.1-1.2 and K-Kv1.2-1.1

**Figure 3:**
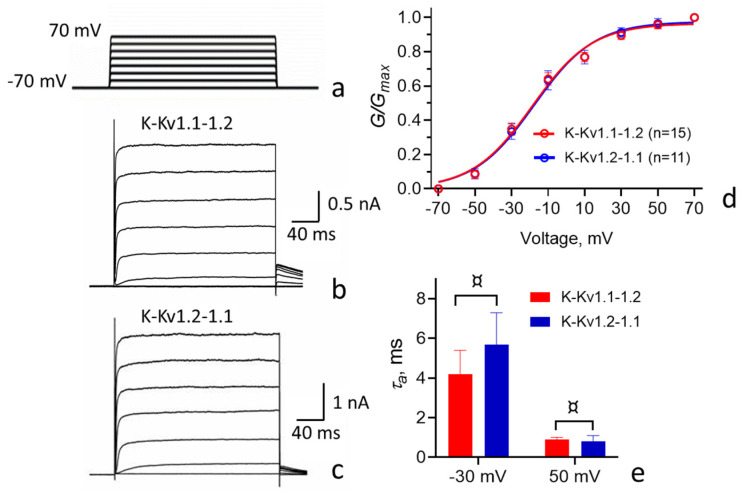
Comparison of the electrophysiological characteristics of the Kv(1.1-1.2)_2_ channels formed in Neuro-2a cells by concatemers K-Kv1.1-1.2 and K-Kv1.2-1.1. (**a**) Protocol of the channel activation. The voltage was varied from −70 mV to +70 mV, with a 20 mV step, followed by returning to a holding potential. Duration of test pulses was 200 ms. The interval between pulses was 5 s. (**b**,**c**) Representative voltage-dependent traces of the outward currents through the channels formed by concatemers K-Kv1.1-1.2 (**b**) and K-Kv1.2-1.1 (**c**). (**d**) Dependences of normalized conductivities (G/G_max_) of the channels formed by concatemers K-Kv1.1-1.2 and K-Kv1.2-1.1 on the applied voltage. (**e**) Rates of the activation (*τ*_a_) of the channels formed by concatemers K-Kv1.1-1.2 and K-Kv1.2-1.1 at the −30 and 50 mV applied voltages (^¤^ *p* > 0.05 according to the unpaired two-tailed *t*-test).

Electrophysiological studies show that K-Kv1.1-1.2 and K-Kv1.2-1.1 embedded in the plasma membrane do form voltage-gated channels (Figure 3a–c).

Comparison of the properties of heterochannels formed by concatemers K-Kv1.1-1.2 or K-Kv1.2-1.1 revealed the close similarity of the character and values of the current through the channels, as well as other characteristics, including the time constant for activation (*τ_a_*) and the dependence of channel conductance on the applied voltage (Figure 3 and Table 1).

The activation threshold for the studied Kv(1.1-1.2)_2_ channels is approximately −40 mV. Channel conductance increases linearly between −40 and 20 mV and saturates above 40 mV. The *τ_a_* values are in the range of several milliseconds at −30 mV and in the sub-millisecond range at 50 mV. The outward current through the channels remains nearly constant in the range from 20 to 200 ms after the application of an opening voltage (between −10 and + 70 mV; Figure 3 and Table 1).

### 3.4. Interactions of HgTx-G and Some Peptide Blockers with Heterochannels

#### 3.4.1. Interactions of HgTx-G with Heterochannels

**Figure 4:**
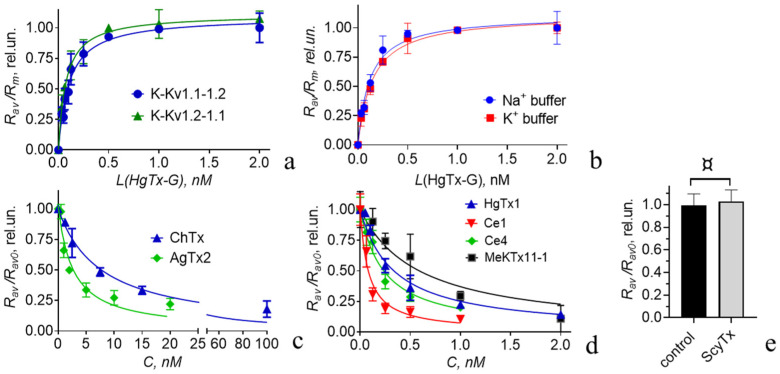
Interactions of HgTx-G and various peptide blockers with Kv(1.1-1.2)_2_ heterochannels formed by the studied concatemers in the membrane of Neuro-2a cells. (**a**) Concentration-dependent binding of HgTx-G to Kv(1.1-1.2)_2_ heterochannels formed by concatemers K-Kv1.1-1.2 and K-Kv1.2-1.1. The measurements were performed in a complete medium. The R_av_/R_m_ ratio characterizes the extent of HgTx-G binding to heterochannels (Equation (1)). (**b**) Concentration dependences of HgTx-G binding to heterochannels Kv(1.1-1.2)_2_ formed by K-Kv1.1-1.2. in a buffer containing either 150 mM KCl (K^+^ buffer, closed channels) or 150 mM NaCl (Na^+^ buffer, open channels). (**c**,**d**) Concentration dependences of the competitive displacement of HgTx-G (0.5 nM) from the complexes with heterochannels Kv(1.1-1.2)_2_ by AgTx2 (**c**), ChTx (**c**), HgTx1 (**d**), Ce1 (**d**), Ce4 (**d**), or MeKtx11-1 (**d**). Heterochannels were formed by K-Kv1.1-1.2. *C* is the concentration of the added peptide pore blocker. The *R_av_*/*R_av0_* ratio characterizes the decrease in HgTx-G binding to the channel as a function of C, as compared to the HgTx-G binding at *C* = 0 (*R_av0_*). Data (**c**,**d**) were averaged over three independent experiments and presented as mean ± SEM. (**e**) Diagram showing that 1 µM ScyTx does not displace 0.5 nM HgTx-G from the complexes with heterochannels Kv(1.1-Kv1.2)_2_ formed by K-Kv1.1-1.2 (mean ± SEM, 3 independent experiments, ^¤^ *p* > 0.05 according to the unpaired two-tailed *t*-test). Control is normalized value R_av0_ (mean ± SEM) for HgTx-G (0.5 nM) bound to Kv(1.1-1.2)_2_ in the absence of ScyTx.

The interaction between HgTx-G and the heterochannels formed by K-Kv1.1-1.2 and K-Kv1.2-1.1 occurs at low-nanomolar and subnanomolar concentrations of HgTx-G (Figure 4a). Concentration dependences of HgTx-G binding to heterochannels formed by K-Kv1.1-1.2 or K-Kv1.2-1.1 are very similar (Figure 4a), and the corresponding values of *K_d_* are equal to 0.10 ± 0.02 and 0.08 ± 0.01 nM (*p* = 0.7953).

Since the order of the α-subunits in the studied concatemers does not affect either the electrophysiological properties of the formed heterochannels (Figure 3 and Table 1) or their ability to bind HgTx-G (Figure 4a), further studies of HgTx-G and peptide blockers were performed on the channels formed by K-Kv1.1-1.2.

**Figure 5:**
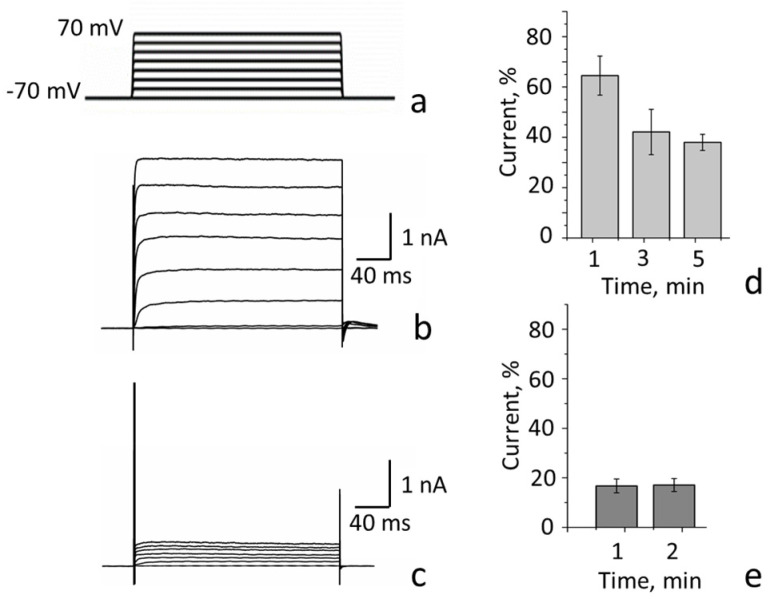
Inhibition of outward currents through Kv(1.1-1.2)_2_ channels in Neuro-2a cells by HgTx-G. The channels were formed by concatemer K-Kv1.1-1.2. (**a**) Protocol of the channel activation. The voltage was varied from −70 mV to +70 mV, with a 20 mV step, followed by returning to a holding potential. Duration of test pulses was 200 ms. The interval between pulses was 5 s. (**b**,**c**) Representative traces of the currents in the absence of HgTx-G (**b**) or in 3 min after application of 10 nM HgTx-G (**c**) at different applied voltages. Inhibition of outward currents was 83 ± 3%. (**d**,**e**) Kinetics of the inhibition of currents through Kv(1.1-1.2)_2_ channels after application of 1 nM (**d**) or 10 nM (**e**) HgTx-G (mean ± SEM). The applied voltage is 70 mV.

HgTx-G efficiently blocks the current through the heterochannels formed by K-Kv1.1-1.2 (Figure 5). The decrease in current through these channels caused by the application of 10 nM HgTx-G is 83 ± 3% when the applied voltage varies from −20 to 70 mV (Figure 5a–c). The residual current is at least partially associated with the basal activity of the voltage-gated channels that are naturally present in Neuro-2a cells and are not sensitive to the blockers of Kv1 channels [34]. The outward currents through these channels can achieve 0.1–0.3 nA [34].

The patch-clamp technique allows one to characterize both efficacy and potency of the blockers. At the same time, accurate measurements of blocker potency at subnanomolar concentrations require a long blocker-application time to reach equilibrium binding. To achieve equilibrium in the binding of 10 nM HgTx-G to the channel, less than 1 min of ligand application is required (Figure 5e), and this time increases to 5 min at 1 nM HgTx-G (Figure 5d). Definitely, the equilibrium time will further increase at lower concentrations of the blocker. The patch-clamp technique is limited in the increase in the blocker application time, which can lead to an underestimation of the blocker affinity in some cases. In contrast, using the fluorescent analyses of the interactions between blockers and channels proposed by us earlier [33,34,35], the time of incubation of blockers with channels expressed on the plasma membrane of cells can vary widely, overcoming the limitation of a patch-clamp technique.

Electrophysiological analysis of the interaction between pore blockers, including HgTx-G, and the channel is performed at the open state of the channel. Fluorescent analysis is usually carried out at the closed state of the channel. To estimate the possible dependence of the affinity of peptide blockers to the channel on the state of the channel, we measured the *K_d_* values of the complexes between HgTx-G and the heterochannel in buffers that induce either the open or closed state of the channel, following the previously proposed procedure [34].

The binding of HgTx-G to the heterochannel is similar in the buffers containing either 150 mM Na^+^ ions (closed state) or 150 mM K^+^ ions (open state) with the *K_d_* values equal to 0.12 ± 0.04 and 0.15 ± 0.03 nM, respectively (Figure 4b). A comparison with the *K_d_* value measured in a complete medium indicates that the components of the cell culture medium and fetal calf serum do not affect the interaction between HgTx-G and the heterochannel.

#### 3.4.2. Interactions of Some Recombinant Peptide Blockers with Heterochannels

Analysis of the Kalium database (https://kaliumdb.org (accessed on 1 July 2025)) shows that data on the interactions of peptide blockers with heterochannels Kv(1.1-1.2)_2_ are very limited. Using cells expressing heterochannels Kv(1.1-1.2)_2_, the fluorescent ligand HgTx-G, and a previously validated method based on the analysis of competitive binding of the fluorescent ligand and the peptide under study to the channel [33,34,35], we characterized the ability of several peptide blockers of Kv1 channels to interact with heterochannels Kv(1.1-1.2)_2_ (Figure 4c–e). The apparent dissociation constants (*K_ap_*) for the peptides studied (HgTx1, AgTx2, ChTx, MeKtx11-1, Ce1, and Ce4) were calculated using the formalism described previously [33] and in Section 2.5. All of these peptides were found to be highly active ligands of the heterochannels Kv(1.1-1.2)_2_ (Table 2). Ce1 has the highest picomolar affinity to the heterochannels Kv(1.1-1.2)_2_ (Figure 4d; Table 2). Ce4 and HgTx1 are approximately threefold less active than Ce1 (Figure 4d; Table 2). MeKtx11-1, AgTx2, and ChTx have approximately 10-, 18-, and 57-fold lower activities, respectively, compared to Ce1 (Figure 4c,d and Table 2). The *K_ap_* for the complexes between Kv(1.1-1.2)_2_ heterochannels and ChTx (Table 2) is similar to that (0.3 nM) determined for similar complexes by the radioligand analysis earlier [41]. In contrast, ScyTx does not compete with 0.5 nM HgTx-G for the binding to heterochannels even at the 1 µM concentration (Figure 4e). It is possible to roughly estimate that, even if ScyTx hypothetically binds to heterochannels Kv(1.1-1.2)_2_, the *K_ap_* of the complex would be higher than 330 nM.

## 4. Discussion

The dimeric concatemers K-Kv1.1-1.2 and K-Kv1.2-1.1 were shown to assemble in the functionally active fluorescent voltage-gated channels with a high presentation in the plasma membrane (Figure 1 and Figure 3). It was found that the properties of these channels, including cellular localization (Figure 1 and Figure 2) and electrophysiological characteristics (Table 1), do not depend on the order of Kv1.1 and Kv1.2 α-subunits in the concatemer. Previous studies of similarly constructed fluorescent concatemers Kv1.1-1.1 and Kv1.2-1.2 have demonstrated that neither the presence of fluorescent protein nor the linker between α-subunits affects the natural electrophysiological characteristics of homotetrameric Kv1.1 and Kv1.2 channels [28,29]. This allows us to suppose that concatemer-based heterochannels Kv(1.1-1.2)_2_ mimic natural heterochannels with the same stoichiometry of the α-subunits. A similar conclusion was made earlier in the studies of heterochannels formed by tetrameric concatemers on the basis of the Kv1.1 and Kv1.2 α-subunits [24,27,28,29]. At the same time, high membrane presentation of heterochannels Kv(1.1-1.2)_2_ was achieved by us artificially by introducing the membrane-targeting mutations S369T and S371T in the Kv1.1 and Kv1.2 α-subunits, respectively [34,35]. In the absence of these mutations, the homotetrameric Kv1.1 and Kv1.2 channels, which were heterologously expressed in Neuro-2a cells, were not detected in the plasma membrane remaining in the cytoplasm [34,35]. Using Förster resonance energy transfer microscopy, it was shown that Kv1.1 and Kv1.2 α-subunits co-expressed in cells formed heterochannels, but it was not a necessary or sufficient condition for the presentation of heterochannels in the plasma membrane [42]. The Kv1.1 α-subunit without S369T mutation blocked membrane transfer of the heterochannels even if the Kv1.2 α-subunit had the membrane-targeting S371T mutation. It was concluded that auxiliary factors, possibly Kvß1 and/or Kvß2 subunits, are necessary for the membrane presentation of native Kv1.1/Kv1.2 heterochannels. Neuro-2a cells do not seem to express these factors in sufficient quantities.

On the basis of our electrophysiological studies and classification proposed earlier [43], we conclude that heterochannels Kv(1.1-1.2)_2_ are low-voltage-activated, highly active, non-inactivating channels with the fast rate of activation (Figure 3; Table 1). They differ from homotetrameric Kv1.2 channels in that they open at a lower membrane potential and have a faster activation rate (Table 1). These electrophysiological properties of Kv(1.1-1.2)_2_ channels are more similar to the properties of the homotetrameric Kv1.1 channel (Table 1). In line, earlier studies of HEK-293 cells expressing rat heterochannels Kv1.1/Kv1.2 formed by tetra-concatenated constructions revealed that the presence of one or two α-subunits Kv1.1 in heterochannels shifted their half-maximum activation voltages to more lower values (V_1/2_ = −20 ± 2 mV), compared to Kv1.2 channels, while V_1/2_ values for rat homotetrameric Kv1.1 and Kv1.2 channels were reported to be −32 ± 1 and −1 ± 1 mV, respectively [27]. The activation rate of rat heterochannels with two α-subunits Kv1.1 (*τ_a_* = 11–14 ms at −20 mV) was more similar to that of the Kv1.1 channel (*τ_a_* = 9 ms) compared to the Kv1.2 channel (*τ_a_* = 20 ms). One can suppose that, in the CNS, membrane expression of heterochannels Kv1.1/Kv1.2 with one or two Kv1.1 α-subunits functionally compensates for the absence of membrane presentation of homotetrameric Kv1.1 channels. 

Designed by us previously [35], HgTx-G was found to bind to Kv(1.1-1.2)_2_ channels and block it (Figure 1 and Figure 5). The measured *K_d_* for the complexes between HgTx-G and Kv(1.1-1.2)_2_ channels (100 pM) is similar to *K_d_* for the complexes between HgTx-G and Kv1.2 or Kv1.3 homotetrameric channels [35]. The *K_d_* for the complexes between HgTx-G and the Kv1.1 homotetrameric channel is four times greater [35]. The affinity of HgTx-G is similar for open and closed heterochannels Kv(1.1-1.2)_2_ (Figure 4b), and it is not compromised by the presence of lipid and protein components in the extracellular medium (Figure 4a). Thus, heterochannels Kv(1.1-1.2)_2_ extend the list of Kv1 channels that can be imaged on the plasma membrane of cells using high-affinity fluorescent ligand HgTx-G. According to our best knowledge, HgTx-G is the first fluorescent ligand, whose properties have been characterized for Kv1 heterochannels.

Our study demonstrates that HgTx-G can also be used as a component of an analytical fluorescence system to reveal the ligands for Kv(1.1-1.2)_2_ channels among peptide toxins (Figure 4) and to measure their affinity for these channels (Table 2).

Using this analytical system, we have studied four well-known and widely used peptide blockers, namely HgTx1, AgTx2, ChTx, and ScyTx, as well as three selective blockers of homotetrameric Kv1.2 channels, namely Ce1, Ce4, and MeKTx11-1.

Data on the lack of interaction between ScyTx and Kv(1.1-Kv1.2)_2_ channels (Figure 4e) highlight the selectivity of ScyTx for small-conductance calcium-activated K+ channels KCa2.2 and KCa2.3 [44].

HgTx1 is a very active inhibitor of Kv1.1, Kv1.2, Kv1.3, and Kv1.6 channels, possessing a nanomolar affinity for Kv1.6 and subnanomolar affinities for Kv1.1, Kv1.2, and Kv1.3, according to the data on the inhibition of ^86^Rb^+^ flux obtained on HEK-293 cells, which heterologously express these channels [14]. Comparison of the activities of HgTx1 using our competitive binding fluorescence-based approach shows it has a very high affinity for heterochannels Kv(1.1-1.2)_2_, similar to its affinity for the homotetrameric Kv1.1 and Kv1.2 channels (Table 2). This result was expected, as the studied heterochannels are composed of α-subunits from two homotetrameric channels that have the same ability to bind this peptide.

AgTx2 is a potent pore blocker of Kv1.1, Kv1.2, Kv1.3, Kv1.6, and Shaker channels [33,34,45,46,47,48]. According to different reports, the peptide affinities for the Kv1.1, Kv1.3, and Kv1.6 channels are either picomolar [45,47,48] or nanomolar [33,34,46]. Affinities of AgTx2 for Kv1.2 and Shaker channels are low nanomolar [46] and subnanomolar [45], respectively. According to our measurements, the *K_ap_* of the complexes between AgTx2 and the Kv(1.1-1.2)_2_ heterochannels is 6- and 86-fold less than that of the complexes with the homotetrameric Kv1.1 and Kv1.2 channels (Table 2). Definitely, the combination of α-subunits from these homotetrameric channels is more favorable for the binding of AgTx2. Our data suggest that there is a narrow range of concentrations (0.25–0.5 nM), when AgTx2 can bind predominantly to the Kv(1.1-1.2)_2_ heterochannels as compared to homotetrameric Kv1.2, Kv1.1, Kv1.3 [33], and probably Kv1.6 [45,46] channels. Thus, AgTx2 may help to study the presence and function of the Kv(1.1-1.2)_2_ heterochannels in cells.

ChTx, Ce1, Ce4, and MeKTx11-1 are peptides that exhibit much higher activity against the Kv1.2 channel than against the Kv1.1 channel (Table 2). Each of them possesses similar activities against both homotetrameric Kv1.2 and heterotetrameric Kv(1.1-1.2)_2_ channels (Table 2). Molecular modeling of the complexes between the Kv1.2 channel and Ce1 or Ce4 peptides revealed that three out of four α-subunits of the channel are involved in interactions with these peptides [35]. One of these three interacting α-subunits participates in peptide binding to a lesser extent than the other two [35]. If non- and weakly interacting α-subunits in the studied heterochannels are Kv1.1 α-subunits, this may partly explain why the presence of these α-subunits does not weaken the interaction between heterochannels Kv(1.1-1.2)_2_ and Ce1 or Ce4 peptides, compared to the interaction between the Kv1.2 channel and the peptides.

In summary, the Ce4 activity against the Kv1.2 and Kv(1.1-1.2)_2_ channels is more than 10,000 and 1000 times higher than the Ce4 activities against Kv1.1 and Kv1.3 channels, respectively (Table 2 and data published previously [35]). Peptides Ce1, MeKTx11-1, and, to a lesser extent, ChTx have similar properties (Table 2 and data published previously [35,38]). This suggests that Ce1, Ce4, and MeKtx11-1 peptides may be used in neuropharmacological studies as molecular tools to selectively suppress the functioning of the Kv1.2 and Kv(1.1-1.2)_2_ channel pools in cells.

## 5. Conclusions

The developed analytical system based on HgTx-G and Neuro-2a cells expressing functionally active fluorescent Kv(1.1-1.2)_2_ channels provides a convenient way to conduct the search for the selective blockers of these channels among known peptide blockers of Kv1 channels or for their design de novo. These studies will also help to extend data on pharmacological profiles of the peptide blockers toward Kv(1.1-1.2)_2_ heterochannels, which are an important component of the nerve impulse conduction mechanism [2]. Also, the cells expressing Kv(1.1-1.2)_2_ channels may be of interest in the development of specific fluorescent ligands for these channels.

Studies of peptides Ce1, Ce4, and MeKTx11-1 have shown that peptides with increased affinity for the Kv1.2 channel can maintain this affinity for Kv(1.1-1.2)_2_ heterochannels. Similarly, high-affinity ligands of Kv1.1 and Kv1.2 channels, such as HgTx1, can also effectively interact with Kv(1.1-1.2)_2_ heterochannels. Interestingly, some peptides, such as AgTx2, which have moderate activity against Kv1.1 and Kv1.2 channels, can interact significantly better with Kv(1.1-1.2)_2_ heterochannels. Further investigation of other peptides and the use of molecular modeling techniques are required to identify the molecular determinants essential for the interaction between Kv(1.1-1.2)_2_ heterochannels and peptide ligands.

## Figures and Tables

**Table 1:** Characteristics of Kv(1.1-1.2)_2_ heterochannels formed by the concatemers K-Kv1.1-1.2 and K-Kv1.2-1.1 and homotetrameric channels Kv1.1 and Kv1.2 formed by α-subunits K-Kv1.1 and K-Kv1.2.

Channel	Kv(1.1-1.2)_2_ (*n* = 14) ^#^	Kv(1.1-1.2)_2_ (*n* = 11)	Kv1.1 * (*n* = 12)	Kv1.2 ** (*n* = 16)
Subunit	K-Kv1.1-1.2	K-Kv1.2-1.1	K-Kv1.1	K-Kv1.2
*τ_a_*(−30 mV ^&^), ms	4.2 ± 1.2	5.7 ± 1.6 ^¤^	4.0 ± 0.5 ^¤^	11 ± 2 ^¶^
*τ_a_*(50 mV), ms	0.89 ± 0.13	0.8 ± 0.3 ^¤^	0.75 ± 0.10 ^¤^	1.2 ± 0.4 ^¤^
*V_1/2_*, mV	−17 ± 2	−16 ± 2 ^¤^	−20 ± 2 ^¤^	−5 ± 2 ^¶^
*k*	18 ± 2	18 ± 2 ^¤^	17 ± 2 ^¤^	17 ± 2 ^¤^

Mean ± SEM values are presented. ^#^ Number of measured cells; * data from [29]; ** data from [28]; ^&^ applied voltage; ^¤^ *p* > 0.05, ^¶^ *p* < 0.005—estimation of the significance of the difference from the corresponding value for Kv(1.1-1.2)_2_ channels formed by K-Kv1.1-1.2 (unpaired two-tailed *t*-test).

**Table 2:** Apparent dissociation constants (*K_ap_*, nM) of the complexes between heterochannels Kv(1.1-1.2)_2_ and studied peptide blockers.

Channel	HgTx1	AgTx2	ChTx	Ce1	Ce4	MeKTx11-1
Kv(1.1-1.2)_2_	0.04 ± 0.01	0.25 ± 0.05	0.8 ± 0.2	0.014 ± 0.006	0.0328 ± 0.0009	0.13 ± 0.05
Kv1.1	0.03 ± 0.02 *^,¤^	1.5 ± 0.7 *^,¤^	>>100 *^,¶^	11 ±5 *^,¶^	>300 ^#,¶^	2110 **
Kv1.2	0.02 ± 0.01 ^#,¤^	21.5 ± 1.2 ^#,¶^	1.0 ± 0.1 ^#,¤^	0.010 ± 0.002 ^#,¤^	0.030 ± 0.010 ^#,¤^	0.19 **

* Data from [34]; ^#^ data from [35]; ** data from [38]; ^¤^ *p* > 0.05; ^¶^ *p* < 0.005—estimation of the significance of the difference from the corresponding value for Kv(1.1-1.2)_2_ channels (unpaired two-tailed *t*-test).

## Data Availability

The data presented in this study are available upon request from the corresponding author. The data are not publicly available due to local regulations.

## Abbreviations

The following abbreviations are used in this manuscript:

AgTx2	agitoxin 2
ChTx	charybdotoxin
CNS	central nervous system
DTX	dendrotoxin
HgTx1	hongotoxin 1
HgTx-G	hongotoxin 1 fused with fluorescent protein GFP
ScyTx	Scyllatoxin
K-Kv1.1	Kv1.1 α-subunit fused with fluorescent protein mKate2
K-Kv1.1-1.2	concatenated dimer Kv1.1-Kv1.2 fused with mKate2
K-Kv1.2	Kv1.2 α-subunit fused with mKate2
K-Kv1.2-1.1	concatenated dimer Kv1.2-Kv1.1 fused with mKate2
Kv(1.1-1.2)_2_	heterochannels formed by concatemers Kv1.1-Kv1.2 fused with mKate2
